# Author Correction: Parkinson’s disease and bacteriophages as its overlooked contributors

**DOI:** 10.1038/s41598-020-69086-9

**Published:** 2020-07-16

**Authors:** George Tetz, Stuart M. Brown, Yuhan Hao, Victor Tetz

**Affiliations:** 1 Human Microbiology Institute, New York, NY 10027 USA; 2 Tetz Laboratories, New York, NY 10027 USA; 3 Applied Bioinformatics Laboratory, NYU School of Medicine, New York, NY 10016 USA

Correction to: *Scientific Reports* 10.1038/s41598-018-29173-4, published online 17 July 2018

This Article contains errors in Figures 3 and 5. Figure 3C incorrectly included a duplicate of Figure 3B. In Figure 5, the bacteriophage Streptococcus 7201, was omitted from the graph.

The correct Figures 3 and 5 appear below as Figures 1 and 2.

**Figure 1:**
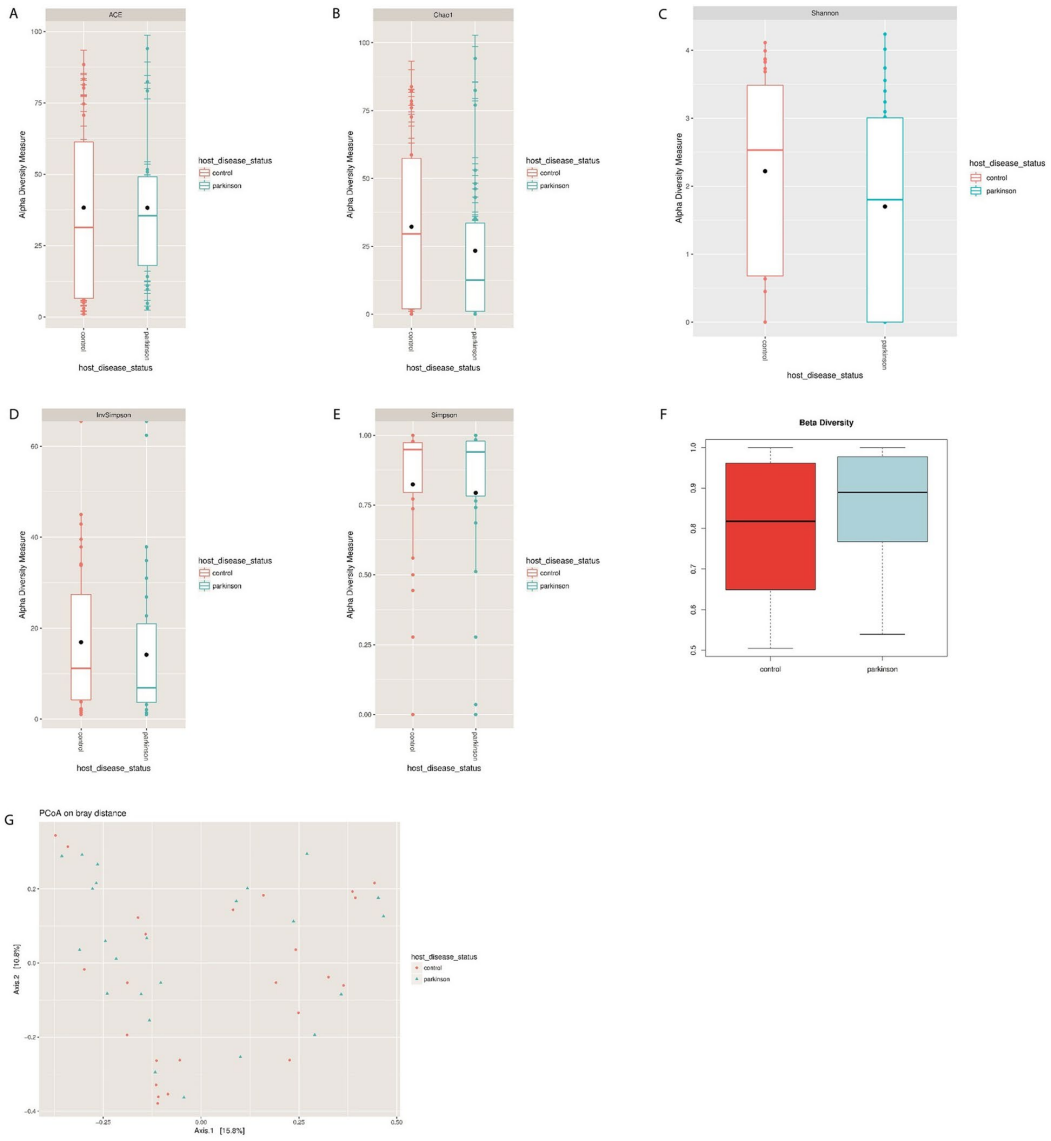
Phagobiome richness in PD patients and healthy individuals. (**A**) ACE, (**B**) Chao1 and α-diversity, (**C**) Shannon, (**D**) Simpson, and (**E**) inverse Simpson indexes. Bacteriophage population diversity in PD patients and healthy individuals. (**F**) β-Diversity of phagobiota was measured using Spearman index. The X axis indicates samples and the Y axis shows Spearman index values: 0.5 means low difference and 1 means high difference (i.e., all species are different) in species diversity between samples. (**G**) PCoA plots of bacteriophage β-diversity based on Bray-Curtis dissimilarity analyses. Each dot represents a scaled measure of the composition of a given sample, colour- and shape-coded according to the group.

**Figure 2:**
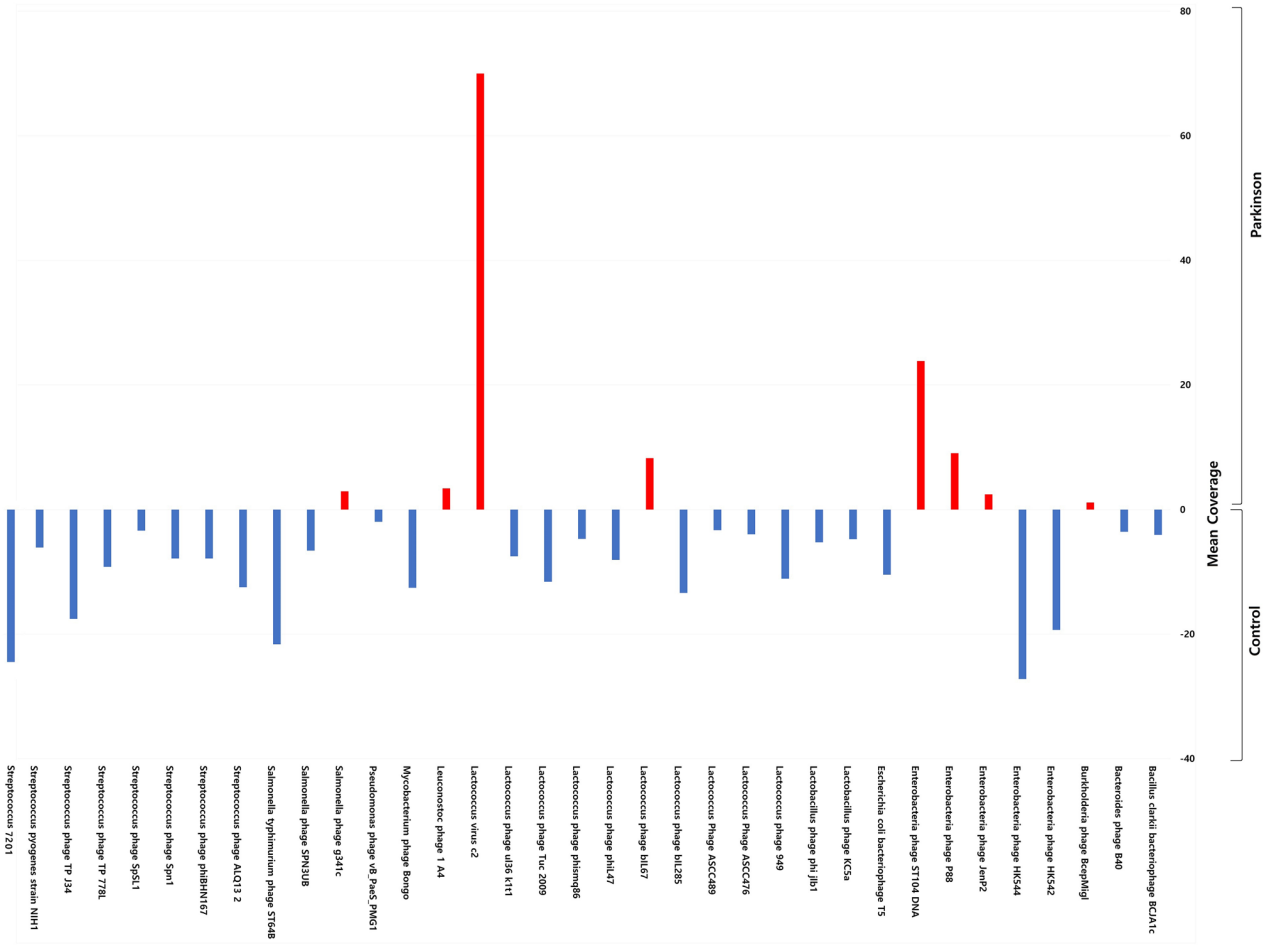
Exploration of bacteriophage diversity in PD patients and healthy participants. The bar graph shows bacteriophage abundance at the genus level in the PD or control group (relative abundance ≥ 0.01% found in at least two samples per group).

