# Supplementary figures and images for: Parkinson’s disease and bacteriophages as its overlooked contributors
Source: Sci Rep. 2018 Jul 17;8:10812. doi: 10.1038/s41598-018-29173-4 (PMC6050259; doi:10.1038/s41598-018-29173-4)

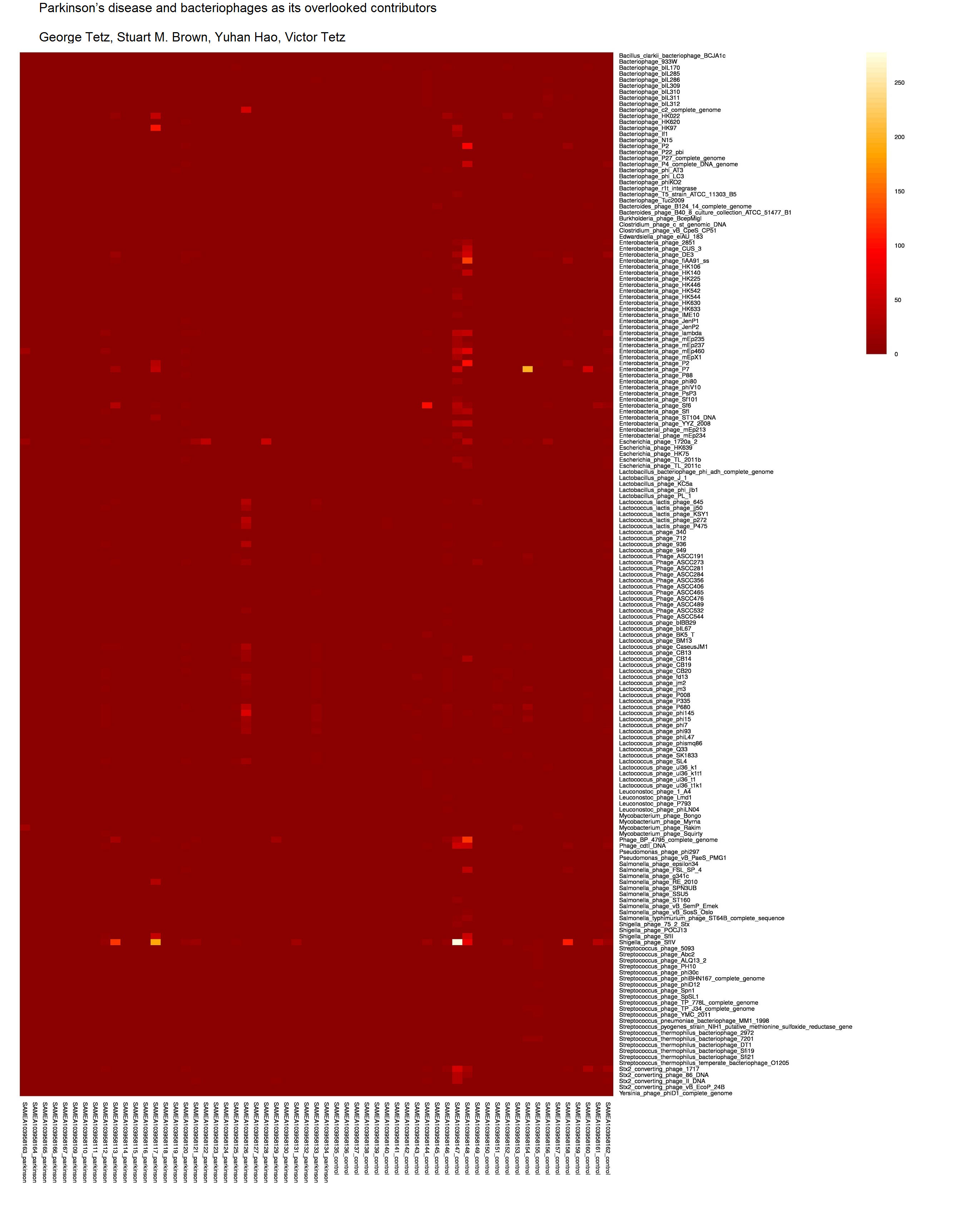

Supplement: Supplementary file 1 — Supplementary Figure S1 [file 41598_2018_29173_MOESM1_ESM.tif]
