# Supplementary material for: Parkinson’s disease and bacteriophages as its overlooked contributors
Source: Sci Rep. 2018 Jul 17;8:10812. doi: 10.1038/s41598-018-29173-4 (PMC6050259; doi:10.1038/s41598-018-29173-4)
Supplement: Supplementary file 8 — Supplementary Table S5. [file 41598_2018_29173_MOESM8_ESM.pdf]

Parkinson’s disease and bacteriophages as its overlooked contributors

George Tetz, Stuart M. Brown, Yuhan Hao, Victor Tetz

Supplementary Table 5*

Clinical characteristics and general demographic parameters of study participants

	**PD**	**Control**	***P* value**
Demographics			
n	31	28	
Age (years, mean ± SD)	64.8 ± 9.5	65.6 ± 10.4	0.970
Clinical data			
UPDRS III (mean ± SD)	12.6 ± 6.9	0 ± 0	<0.001
GIT symptoms incl. constipation (GSRS, mean ± SD)	3.4 ± 2.9	2.2 ± 2.0	0.172
Total serum bilirubin	0.23 ± 0.03	0.23 ± 0.03	0.593
Nutritional habits			
Diet			
Omnivorous	30 [96.8%]	28 [100%]	
Vegetarian	1 [3.2%]	0 [0%]	
Probiotics	4 [12.9%]	1 [3.6%]	
Medication			
Amantadine	26 [83.9%]	0 [0%]	
Dopamine agonist	11 [35.5%]	0 [0%]	
MAO inhibitor	28 [90.3%]	0 [0%]	
L-DOPA	0 [0%]	0 [0%]	
Statin intake	1 [3.2%]	11 [39.3%]	
Metformin	1 [3.2%]	3 [10.7%]	
Acetylsalicylic acid	2 [6.5%]	7 [25.0%]	
Smoking			
No	10 [32.3%]	9 [32.1%]	
Yes	5 [16.1%]	4 [14.3%]	
Ex-smoker	15 [48.4%]	15 [53.6%]	

**Originally from the work of Bedarf et al. 2016*

UPDRS Unified Parkinson’s Disease Rating Scale, GIT gastrointestinal, GSRS Gastrointestinal Symptoms Rating Scale, MAO monoamine oxidase, L-DOPA Levodopa
